# Hypercapnia Increases Influenza A Virus Infection of Bronchial Epithelial Cells by Augmenting Cellular Cholesterol via mTOR and Akt

**DOI:** 10.3390/ijms26094133

**Published:** 2025-04-26

**Authors:** Fei Chen, Aiko Matsuda, Peter H. S. Sporn, S. Marina Casalino-Matsuda

**Affiliations:** 1 Division of Pulmonary and Critical Care Medicine, Feinberg School of Medicine, Northwestern University, Chicago, IL 60611, USA; 2 Research Service, Jesse Brown Veterans Affairs Medical Center, Chicago, IL 60612, USA

**Keywords:** hypercapnia, influenza A virus, cholesterol, mTOR, Akt

## Abstract

Hypercapnia, the elevation of CO_2_ in blood and tissue, is a risk factor for mortality in patients with severe lung disease and pulmonary infections. We previously showed that hypercapnia increases viral replication and mortality in mice infected with influenza A virus (IAV). Elevated CO_2_ also augmented cholesterol content and pseudo-SARS-CoV-2 entry in bronchial epithelial cells. Interestingly, cellular cholesterol facilitates IAV uptake, replication, assembly, and egress from cells. Here, we report that hypercapnia increases viral protein expression in airway epithelium of mice infected with IAV. Elevated CO_2_ also enhanced IAV adhesion and internalization, viral protein expression, and viral replication in bronchial epithelial cells. Hypercapnia increased the expression and activation of the transcription factor sterol-regulatory element binding protein 2 (SREBP2), resulting in elevated expression of cholesterol synthesis enzymes, decreased expression of a cholesterol efflux transporter, and augmented cellular cholesterol. Moreover, reducing cellular cholesterol with an SREBP2 inhibitor or statins blocked hypercapnia-induced increases in viral adhesion and internalization, viral protein expression, and IAV replication. Inhibitors of mTOR and Akt also blocked the effect of hypercapnia on viral growth. Our findings suggest that targeting cholesterol synthesis and/or mTOR/Akt signaling may hold promise for reducing susceptibility to influenza infection in patients with advanced lung disease and hypercapnia.

## 1. Introduction

Hypercapnia, elevation of the partial pressure of carbon dioxide (CO_2_) in blood and tissue, commonly develops in severe acute and chronic lung diseases, including advanced chronic obstructive pulmonary disease (COPD) [1,2,3,4,5]. Importantly, these clinical scenarios associated with hypercapnia all carry a high risk of pulmonary infection, including community-acquired pneumonia [6]. Also, bacterial and viral pulmonary infections, especially influenza [7,8,9], are a principal cause of acute COPD exacerbations [10] and are linked to increased need for hospitalization and to mortality [11]. Moreover, hypercapnia is an independent risk factor for mortality in adults hospitalized with community-acquired pneumonia [6,12], children with adenoviral lung infections [13], and patients with cystic fibrosis awaiting lung transplantation [14]. In addition, in patients with acute respiratory failure due to severe COVID-19, hypercapnia was associated with prolonged time on mechanical ventilation and a greater length of stay in the intensive care unit [15]. We previously reported that hypercapnia increased expression of viral proteins, viral replication, lung injury, and mortality in mice infected with influenza A virus (IAV) [16,17]. Also, in a transcriptional profiling study of differentiated human bronchial epithelial (HBE) cells, we found that hypercapnia increased expression of cholesterol biosynthesis genes, including 3-hydroxy-3-methylglutaryl-CoA synthase 1 (*HMGCS1*), and decreased expression of *ABC* transporters, which promote cholesterol efflux [18]. These genes are regulated by the transcription factor sterol-regulatory element binding protein 2 (SREBP2), master regulator of cholesterol synthesis and transport gene expression [19]. Further, we reported that hypercapnia increases protein expression and activation of SREBP2, elevating cellular cholesterol and enhancing entry of SARS-CoV-2 pseudovirus into bronchial epithelial cells [20]. Previous findings that cellular cholesterol is also important for binding, internalization, and budding of influenza virus [21,22,23,24] suggested that hypercapnia might enhance IAV infection by this pathway as well. In another study, we showed that hypercapnia increases Akt phosphorylation and that inhibition of Akt blocks hypercapnia-induced IAV replication in alveolar macrophages [16]. Notably, Akt functions both upstream and downstream in the mTOR pathway, which plays a central role in regulating translation, lipid synthesis, nucleotide synthesis, lysosome biogenesis, nutrient sensing, and growth factor signaling [25]. mTOR signaling is mediated by two distinct multi-subunit complexes, mTOR complex 1 and 2 (mTORC1 and mTORC2), which incorporate the proteins Raptor and Rictor, respectively, as well as other binding partners. Akt activates mTORC1 by phosphorylating and deactivating its negative regulator, Tsc2 [26]. Activated mTORC1, in turn, phosphorylates and activates ribosomal S6 kinases S6K1 and S6K2, while mTORC2 phosphorylates and activates Akt [25,26]. Whether hypercapnia activates Akt in epithelial cells, and the possibility that mTOR and Akt mediate effects of elevated CO_2_ in bronchial epithelium, has not previously been investigated.

In the current study, we explored the effects of hypercapnia on IAV infection in airway epithelial cells. We found that hypercapnia increases viral protein expression in mouse airway epithelium and human bronchial epithelial cells. Elevated CO_2_ also augmented cellular cholesterol content, and decreasing cellular cholesterol with statins or an SREBP2 inhibitor blocked the hypercapnia-induced elevation in IAV adhesion and internalization, viral protein expression, and viral replication in epithelial cells. These effects of hypercapnia were dependent on CO_2_-induced signaling via mTOR and Akt, which, in turn, increased expression and activation of SREBP2. Our data suggest that targeting cholesterol synthesis and/or mTOR/Akt signaling may hold promise for ameliorating hypercapnia-induced immunosuppression and improving resistance to viral infection in patients with advanced lung disease and hypercapnia.

## 2. Results

### 2.1. Hypercapnia Increases Viral Protein Expression in Mouse Bronchial Epithelium Following IAV Infection In Vivo and in Human Bronchial Epithelial Cells Infected with IAV In Vitro

**Figure 1:**
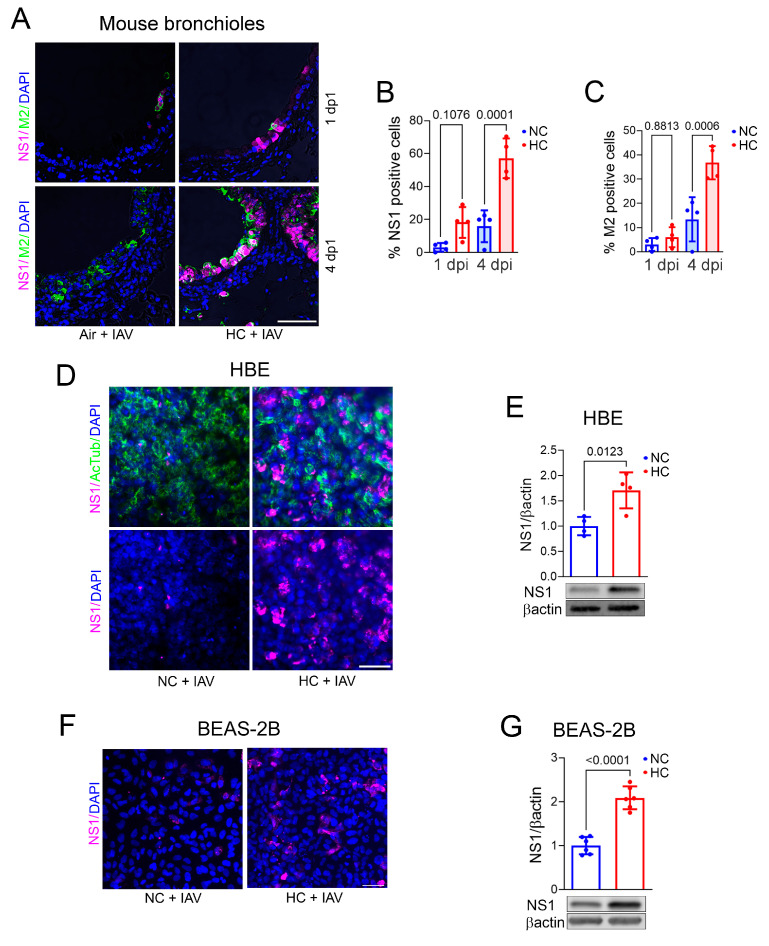
Hypercapnia increases viral protein expression in mouse bronchial epithelium, human bronchial epithelial (HBE), and BEAS-2B cells infected with IAV. Mice breathing 10% CO_2_/21% O_2_ (hypercapnia, HC), or air as control, were infected with 30 pfu IAV and then sacrificed 1 and 4 dpi. Expression of viral NS1 (magenta) and M2 in lung tissue was assessed by IF microscopy (**A**). NS1 (**B**) and M2 (**C**) were quantified as percentage of NS1- or M2-positive cells. Airways (3–4) from lungs of 4 different mice from 2 independent experiments were analyzed. *p-*values from ANOVA followed by Sidak’s multiple comparisons test (**B**,**C**) are shown. HBE cells differentiated at air–liquid interface (ALI) and BEAS-2B cells were cultured under normocapnic (NC, 5% CO_2_/95% air) or hypercapnic (HC, 15% CO_2_/21% O_2_/64% N_2_) conditions for 2 days, infected with IAV (MOI 1) cultured for an additional day in NC or HC, respectively, and then fixed. Expression of viral NS1 (**D**,**F**) and acetylated tubulin (a-Tub, marker of airway ciliated cells) were assessed by IF. Scale bars = 50 µm (**A**,**D**,**F**). NS1 expression was also assessed by immunoblot, with βactin as loading control (**E**,**G**); individual data points, means ± SEM of arbitrary density units from N = 4 (**E**) and N = 6 (**G**) independent experiments, and *p-*values for comparison of NC and HC using Student’s *t*-test (**E**,**G**) are shown.

To investigate the effects of elevated CO_2_ on IAV infection in vivo, we exposed mice to normoxic hypercapnia (10% CO_2_/21% O_2_) for 3 days prior to virus infection. We previously reported that exposure of mice to 10% CO_2_ for 3 days increases arterial PCO_2_ to ~75 mm Hg, as compared to ~40 mm Hg in air-breathing animals, and allows for maximal renal compensation of respiratory acidosis, resulting in an arterial pH of ~7.3 [27]. Mice were then infected with 30 plaque-forming units (pfus) IAV (A/WSN/1933), after which expression of viral proteins in lung tissue was assessed by immunofluorescence (IF) microscopy. As shown in Figure 1A–C, expression of IAV non-structural protein 1 (NS1), which inhibits the host antiviral response and regulates viral replication [28], and matrix-2 protein (M2), a proton channel essential for the virus to replicate [29], was elevated in bronchial epithelium of mice breathing 10% CO_2_, as compared to air, at 4 days postinfection (dpi). Similarly, NS1 protein expression was elevated in HBE (Figure 1D,E) and BEAS-2B (Figure 1F,G) cells infected with IAV when cultured under hypercapnic (15% CO_2_/21% O_2_/64% N_2_) as compared to normocapnic (5% CO_2_/95% air) conditions. These results corroborate our previous finding that hypercapnia increases IAV infection of mouse bronchial epithelium in vivo [16] and demonstrate that elevated CO_2_ increases IAV protein expression and replication in human bronchial epithelial cells infected with IAV in vitro.

### 2.2. Hypercapnia Increases Cellular Cholesterol, and Inhibition of the Transcription Factor SREBP2 Blocks Hypercapnia-Induced Changes in Expression of the Cholesterol Synthesis Enzyme HMGCS1 and Efflux Transporter ABCA1

**Figure 2:**
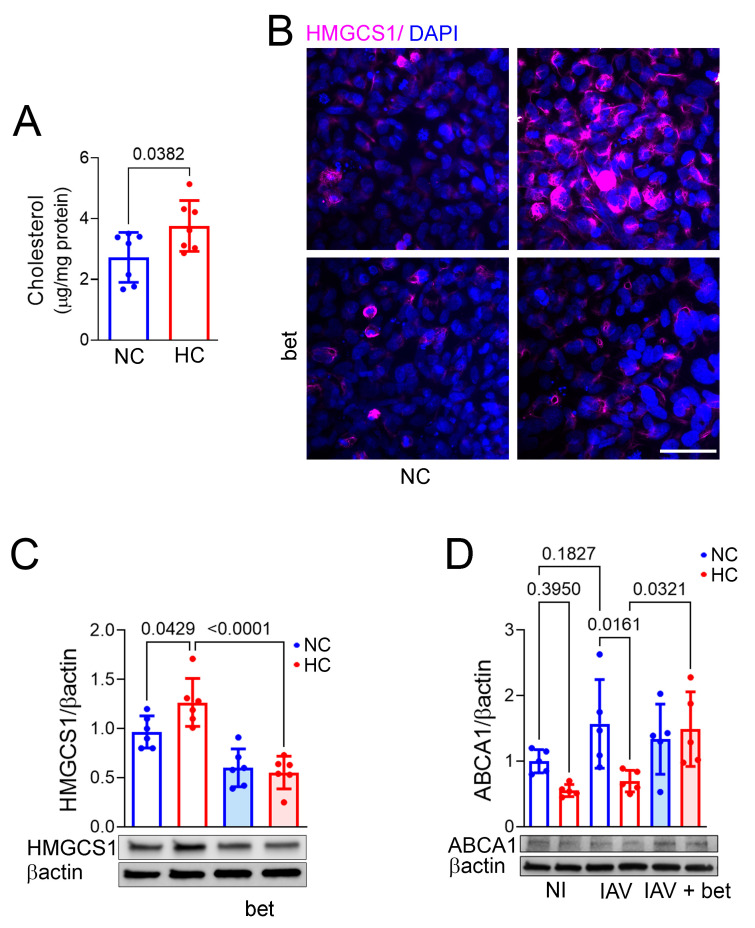
Hypercapnia increases cellular cholesterol, and inhibition of SREBP2 blocks hypercapnia-induced changes in expression of HMGCS1 and ABCA1 proteins. BEAS-2B cells were cultured in NC (5% CO_2_/95% air) or HC (15% CO_2_/21% O_2_/64% N_2_) for 4 days, after which cellular cholesterol was measured using Amplex Red assay (**A**). Alternatively, BEAS-2B cells pre-treated with the SREBP2 inhibitor betulin (bet, 7.5 µM) or vehicle were cultured in NC or HC for 3 days and then immunostained and assessed for HMGCS1 (magenta) by IF microscopy (**B**) or immunoblot, N = 4 independent experiments (**C**). Nuclei were stained with DAPI; scale bar = 50 µm (**B**). BEAS-2B cells pre-treated with betulin or vehicle and cultured in NC or HC were also infected with IAV (MOI 1) or not infected as control (NI), and 1 day later, cells were lysed and immunoblotted for ABCA1 with βactin as loading control, N = 5 independent experiments (**D**). *p*-values from Student’s *t*-test (**A**) or ANOVA followed by Sidak’s multiple comparisons test (**C**,**D**) are shown.

Cholesterol in the cell membrane and intracellular compartments plays a key role in influenza virus binding, internalization, and budding [21,22,23,24]. We previously showed that hypercapnia increases expression and activation of SREBP2, which regulates transcription of cholesterol synthesis and transport genes, concomitantly increasing expression of the key cholesterol synthesis enzyme HMGCS1, and decreased cholesterol efflux transporter ABCA1, in mouse bronchial epithelium [20]. Here we show that culture under hypercapnic conditions augmented the cholesterol content of BEAS-2B cells (Figure 2A). In addition, we found that the SREBP2 inhibitor betulin blocked the CO_2_-induced elevation in HMGCS1 protein expression in these cells (Figure 2B,C). Moreover, betulin blocked the decrease in ABCA1 caused by hypercapnia in BEAS-2B cells infected with IAV (Figure 2D). Thus, hypercapnia increases the cholesterol content of epithelial cells, which enhances their susceptibility to infection by IAV. The CO_2_-induced increase in cholesterol is accompanied by augmented expression of the cholesterol synthesis enzyme HMGCS1 and decreased expression of the efflux transporter ABCA1 ([20] and Figure 2B–D). Hypercapnia also increases expression of SREBP2, and an inhibitor of the transcription factor blocks the hypercapnia-induced changes in expression of both HMGCS1 and ABCA1.

### 2.3. Inhibiting Cellular Cholesterol Accumulation Blocks Hypercapnia-Induced Increases in IAV Adhesion and Internalization, NS1 Expression, and Viral Replication in Bronchial Epithelial Cells

**Figure 3:**
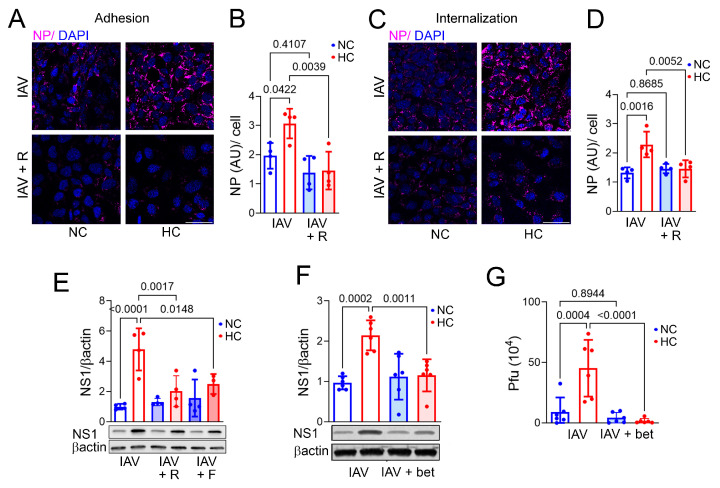
Inhibition of cellular cholesterol accumulation blocks hypercapnia-induced increases in adhesion, internalization, NS1 expression, and replication of IAV in bronchial epithelial cells. BEAS-2B cells cultured in NC (5% CO_2_/95% air) or HC (15% CO_2_/21% O_2_/64% N_2_) were pre-treated with rosuvastatin (R, 0.5 µM), fluvastatin (F, 50 nM), betulin (bet, 7.5 µM), or vehicle prior to addition of IAV. To assess viral adhesion, cells were placed on ice before adding IAV (MOI 20); after 90 min on ice, cells were washed with ice-cold media and fixed. To assess viral internalization, cells were placed on ice before adding IAV (MOI 20); after 90 min on ice, cells were returned to NC or HC at 37 °C for 30 min and then washed and fixed. Adhesion (**A**) and internalization (**C**) were assessed by IF microscopy of cells stained for NP (magenta); nuclei were stained with DAPI. Scale bars = 50 µm (**A,C**). NP was quantified as corrected total cell fluorescence and expressed in arbitrary units (AU)/cell, for adhesion (**B**) and internalization (**D**), N = 4 independent experiments. In other experiments, BEAS-2B cells cultured in NC or HC and maintained at 37 °C throughout were infected with IAV (MOI 1), and 1 day later, cells were lysed for immunoblotting, or culture supernatants were removed for plaque assay. NS1 expression was assessed by immunoblot with βactin as loading control, N = 4 independent experiments (**E**), and N = 6 independent experiments (**F**). Viral titers in culture supernatants were determined by plaque assay and expressed as pfu, N = 6 independent experiments (**G**). Individual data points from independent experiments, means ± SEM, and *p*-values for comparison of NC or HC using ANOVA followed by Sidak’s multiple comparisons test are shown (**B**,**D**–**G**).

The initial steps in IAV infection include binding to the surface of a target cell, internalization, and trafficking of the virus within the cell. Cholesterol in the target cell membrane plays a critical role in each of these processes [23,30]. Therefore, we assessed the impact of hypercapnia on IAV adhesion and internalization in BEAS-2B cells. We found that hypercapnia increased both IAV adhesion (Figure 3A,B) and internalization (Figure 3C,D), assessed by quantitative analysis of cells immunostained for viral nucleoprotein (NP), which is critical for IAV nuclear import, genome transcription, and assembly [31]. To evaluate the possibility that hypercapnia enhanced viral adhesion and entry by increasing cholesterol in the cell membrane, we used statins which inhibit the rate-limiting cholesterol synthesis enzyme 3-hydroxy-3-methyl glutaryl-CoA reductase (HMGCR) [32,33] and the SREPB2 inhibitor betulin. We found that rosuvastatin blocked the increases in IAV adhesion (Figure 3A,B) and internalization (Figure 3C,D) induced by elevated CO_2_. In addition, rosuvastatin and fluvastatin blocked the hypercapnia-induced elevation in NS1 expression in BEAS-2B cells (Figure 3E). Betulin also blocked the CO_2_-induced increase in NS1 expression (Figure 3F) and the increase in IAV viral titer (Figure 3G) induced by hypercapnia. Thus, blocking the CO_2_-induced increase in cellular cholesterol prevents enhanced IAV adhesion and internalization, NS1 expression, and viral replication in bronchial epithelial cells cultured in hypercapnia.

### 2.4. Inhibition of mTOR Signaling Blocks the Hypercapnia-Induced Increase in Activation of SREBP2, Viral Protein Expression, and IAV Replication

**Figure 4:**
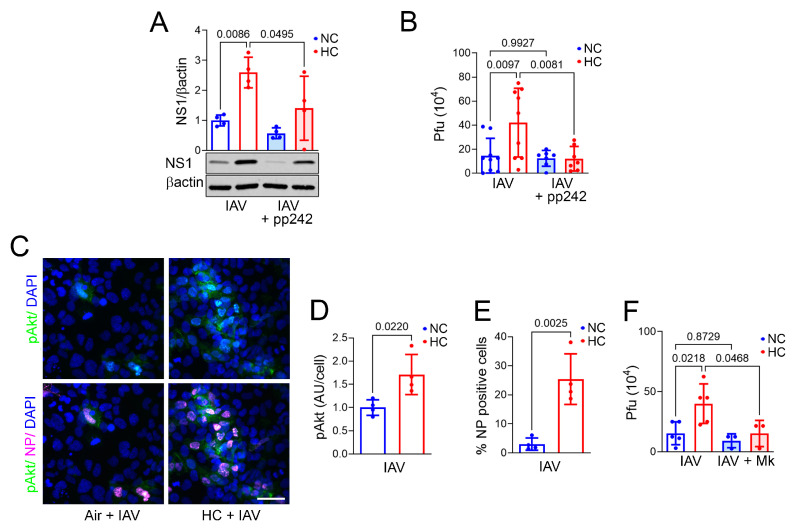
Inhibition of mTOR signaling and Akt blocks hypercapnia-induced increases in NS1 protein expression and IAV replication. BEAS-2B cells cultured in NC (5% CO_2_/95% air) or HC (15% CO_2_/21% O_2_/64% N_2_) were pre-exposed to the dual mTORC1/mTORC2 inhibitor pp242 (1 µM), the Akt inhibitor Mk2206 (Mk, 5 µM), vehicle (**A**,**B**,**F**), or no inhibitor (**C**–**E**) and then infected with IAV (MOI 1) and cultured for an additional day. Cells were then lysed for immunoblotting, fixed for IF microscopy, or supernatants were removed for plaque assay. NS1 protein was assessed by immunoblot with βactin as loading control, N = 4 independent experiments (**A**). Phosphorylated Akt (pAkt, green) and viral NP (magenta) were assessed by IF microscopy, scale bar = 50 µm (**C**). pAkt was quantified as corrected total cell fluorescence and expressed in arbitrary units (AUs)/cell (**D**) and NP as a percentage of NP-positive cells (**E**), N = 4 independent experiments. Viral titers in culture supernatants were determined by plaque assay and expressed as pfu, N = 8 (**B**) and N = 5 independent experiments (**F**). Individual data points, means ± SEM, and *p*-values using ANOVA followed by Sidak’s multiple comparisons test (**A**,**B**,**F**) or Student’s *t*-test (**D**,**E**) are shown.

As noted, we previously showed that hypercapnia activates Akt in macrophages and that inhibition of Akt blocks hypercapnia-induced viral replication in alveolar macrophages [16]. mTOR plays a central role in regulating translation, lipid synthesis, nucleotide synthesis, biogenesis of lysosomes, nutrient sensing, and growth factor signaling [34]. mTOR signaling is mediated by two distinct multi-subunit complexes, mTOR complex 1 and 2 (mTORC1 and mTORC2), which incorporate the proteins Raptor and Rictor, respectively, as well as other binding partners. Major downstream targets activated by mTORC1 include ribosomal S6 kinases S6K1 and S6K2, while mTORC2 activates Akt [34]. In turn, S6K1 and Akt each activate SREBP2 [35,36,37]. In addition, we have previously published the finding that hypercapnia increases SREBP2 activation in BEAS2B cells [20]. Thus, we investigated the effect of the dual mTORC1/mTORC2 inhibitor pp242 [25,38] on SREPB2 activation in IAV-infected BEAS-2B cells. We found that pp242 blocked the hypercapnia-induced increase in the cleaved SREBP2 isoform (Figure S1), which is released from the endoplasmic reticulum and translocates to the nucleus. In addition, pp242 blocked hypercapnia-induced increases in cellular cholesterol (Figure S2), viral NS1 protein (Figure 4A), and viral replication (Figure 4B). Thus, blocking signaling via mTORC1 and mTORC2 inhibits activation of SREBP2, which decreases cellular cholesterol, thereby preventing the increase in IAV replication induced by elevated CO_2_.

### 2.5. Hypercapnia Triggers mTOR Signaling, Which Activates S6K1 and Akt in IAV-Infected Bronchial Epithelial Cells

We previously showed that hypercapnia increases Akt phosphorylation and that inhibition of Akt blocks CO_2_-induced viral replication in alveolar macrophages [16]. Notably, Akt is known to function both upstream and downstream in the mTOR pathway, in that it can activate mTORC1 and is, in turn, activated by both mTORC1 and mTORC2 [34]. Thus, we investigated the impact of the Akt inhibitor Mk2206 and the mTORC1/mTORC2 inhibitor pp242 on phosphorylation of the mTORC1 downstream substrate S6K1 and the mTORC2 target Akt [39]. First, we showed that hypercapnia increased activation of both S6K1 (Figure S3A) and Akt in IAV-infected BEAS-2B cells (Figure S3A and Figure 4C,D). CO_2_-induced activation of S6K1 phosphorylation in IAV infected cells was blocked by pp242 but not MK2206 (Figure S3A). On the other hand, Akt phosphorylation induced by hypercapnia in infected cells was blocked by both pp242 and Mk2206 (Figure S3B). These results suggest that hypercapnia activates mTORC1 and mTORC2, that activation of mTORC1 is independent of Akt, and that CO_2_-induced activation of Akt t is mediated by mTOR signaling via mTORC2.

### 2.6. Inhibition of Akt Blocks Hypercapnia-Induced Increases in IAV Replication and HMGCS1 Expression in BEAS-2B Cells and Blocks HMGCS1 and SREBP2 Expression in Mouse Bronchial Epithelium

**Figure 5:**
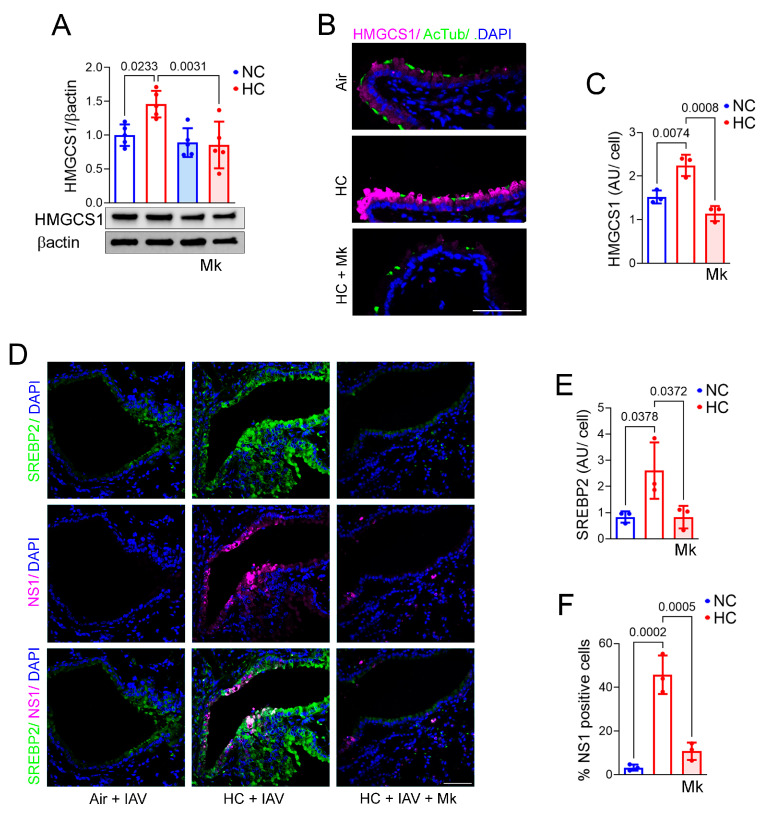
Inhibition of Akt blocks hypercapnia-induced increases in expression of HMGCS1 in BEAS-2B cells and HMGCS1, SREBP2, and viral NS1 in bronchial epithelium of IAV-infected mice. BEAS-2B cells cultured in NC (5% CO_2_/95% air) or HC (15% CO_2_/21% O_2_/64% N2) were pre-exposed to the Akt inhibitor Mk2206 (Mk, 5 µM) or vehicle for 2 h. One day later, cells were lysed and immunoblotted for HMGCS1 with βactin as loading control (**A**). Mice were exposed to 10% CO_2_/21% O_2_ or air as control. MK2206 (120 mg/kg body weight) or the drug vehicle captisol was administrated by oral gavage at 1 d before HC exposure and at 2 days after HC exposure. Mice were sacrificed after 7 days of HC exposure (**B**) or infected with IAV, as before, after 3 days of HC exposure and sacrificed at 7 dpi (**D**). Expression of HMGCS1 (magenta) and acetylated tubulin (**B**); SREBP2 and viral NS1 (magenta) (**D**) were assessed using IF microscopy; nuclei were stained with DAPI (blue); scale bars = 50 µm. HMGCS1 (**C**) and SREBP2 (**E**) protein expression was quantified and expressed as AU/cell. NS1 (**F**) was quantified as percentage of NS1-positive cells. Airways (3–4) from lungs of 3 different mice from 3 independent experiments were analyzed. Individual data points, means ± SEM, and *p* values for comparisons of NC or HC using ANOVA followed by Sidak’s multiple comparisons test (**C**,**E**,**F**) are shown.

In addition to showing that hypercapnia activates Akt in IAV-infected BEAS-2B cells, we confirmed that this was associated with increased expression of viral NP protein in the setting of elevated CO_2_ (Figure 4C,E). Furthermore, the Akt inhibitor Mk2206 [40] blocked the hypercapnia-induced elevation in IAV replication (Figure 4F) and expression of HMGCS1 (Figure 5A) in BEAS-2B cells. This result parallels our previous finding that Mk2206 inhibited IAV replication in macrophages cultured in hypercapnia [16]. To determine the in vivo relevance of our findings in cultured BEAS-2B cells, we assessed expression of HMGCS1 and SREBP2 in the bronchial epithelium of mice exposed to air or 10% CO_2_ and dosed with Mk2206 by oral gavage. We found that Mk2206 blocked a robust hypercapnia-induced increase in bronchial epithelial HMGCS1 expression in the absence of infection (Figure 5B,C) and prevented increases in expression of SREBP2 and viral NS1 expression in IAV-infected mice breathing elevated CO_2_ (Figure 5D–F). These results suggest that activation of Akt by hypercapnia drives augmented expression of SREBP2 and HMGCS1, which increases cellular cholesterol, thereby facilitating IAV infection and increasing viral replication in bronchial epithelium.

## 3. Discussion

In the current study, we show for the first time that hypercapnia increases IAV infection in mouse and human bronchial epithelium, where influenza viruses first interact with the respiratory system and replicate [41]. We found that hypercapnia augmented viral adhesion and internalization, viral protein expression, and viral replication in bronchial epithelial cells. Hypercapnia also increased cellular cholesterol content, which occurred in association with elevated expression and activation of the transcription factor SREPB2 and the cholesterol synthesis enzyme HMGCS1 and with decreased expression of the cholesterol efflux transporter ABCA1. Moreover, pharmacological inhibition of cholesterol accumulation using betulin to inhibit SREBP2 or statins to inhibit HMGCR blocked the CO_2_-induced increases in IAV adhesion and internalization, viral proteins expression, and viral replication. In addition, we found that hypercapnia activates both mTOR signaling and Akt, that the dual mTORC1/mTORC2 inhibitor pp242 and the Akt inhibitor Mk2206 block increased expression and activation of SREBP2 (thereby preventing the elevation in cellular cholesterol), and that Mk2206 blocks NS1 protein expression (a correlate of IAV replication) in bronchial epithelial cells. Taken together, these results establish a causal role for mTOR and Akt signaling leading to an SREBP2-mediated increase in cellular cholesterol as the mechanism underlying the hypercapnia-induced elevation in IAV infection in bronchial epithelial cells.

Our observation that hypercapnia increased expression and activation of SREBP2, augmented HMGCS1, decreased ABCA1 expression, and increased cellular cholesterol content recapitulates similar findings in our previous study of hypercapnia’s effect on uptake of pseudo-SARS-CoV-2 in human bronchial epithelial cells [20]. By regulating cholesterol metabolism genes and increasing cellular cholesterol [19,42], SREBP2 had previously been shown to promote replication of IAV and other viruses [43,44,45]. Entry of influenza virus into host cells, virion assembly, and viral budding are dependent on the presence of cholesterol and lipid rafts in the host cells’ membranes [23,46]. Through its outer surface protein hemagglutinin, IAV binds sialic acid residues in glycolipids that concentrate with cholesterol in lipid rafts [47,48]. Moreover, cholesterol in the target membrane enhances viral binding avidity in a concentration-dependent manner [21]. These mechanisms likely contribute to the CO_2_-induced increase in viral replication in bronchial epithelial cells infected with IAV in hypercapnia. The elevation in viral growth was accompanied by increased expression of the IAV-encoded proteins NS1, NP, and M2 in epithelial cells exposed to elevated CO_2_ in vitro or in vivo; each of these proteins plays a critical role in the ability of the virus to replicate [28,29,31,49].

Given the importance of cholesterol in IAV adhesion and internalization, our finding that pharmacological inhibition of SREBP2 with betulin or HMGCR with statins blocks the hypercapnia-induced increase in IAV growth in bronchial epithelial cells strongly suggests that the increase in cellular cholesterol induced by elevated CO_2_ is central to the effect of hypercapnia on worsening outcomes of IAV infection in mice. However, our previous observation that hypercapnia suppresses the interferon-mediated antiviral host response to IAV without affecting virus adhesion or internalization in macrophages [16] indicates that elevated CO_2_ augments viral replication by more than one mechanism and that the pathways involved may differ depending on cell type.

Independent of hypercapnia, some reports indicate that pharmacologic inhibition or genetic silencing of SREBP2 [43,44,50,51] or statins suppresses viral infection [52], while others show no benefit of statins on IAV infection [53,54]. Here we show that betulin and statins inhibit IAV infection in bronchial epithelial cells under hypercapnia but not normocapnia. This suggest that conflicting results of clinical studies of statins on influenza outcomes [54,55] might, in part, be explained by the fact that none of these studies stratified patients according to whether or not they were hypercapnic.

SREBP2 is activated by several pathways, including the mTOR signaling pathway [37]. Activation of SREBP2 is mediated by mTORC1, which directly phosphorylates the ribosomal protein S6K1 [37,56]. Interestingly, the SREBP2 inhibitor betulin, which blocked the hypercapnia-induced increases in viral protein expression and IAV replication, has also been known to inhibit the mTOR pathway [57]. In addition, the PI3K/Akt/mTORC1 pathway is involved in SREBP2 transport to the Golgi, where it undergoes cleavage and activation [36,58]. It has also been reported that IAV activates mTORC1 and mTORC2 signaling and that the viral NS1 protein specifically promotes phosphorylation of Akt at a distinct site via mTORC2 [59]. In our study, the hypercapnia-induced increase in SREPB2 activation in IAV-infected bronchial epithelial cells was blocked by pp242, which inhibits both mTORC1 and mTORC2 [38]. This inhibitor also blocked increases in NS1 expression and IAV proliferation under conditions of elevated CO_2_. On the other hand, the Akt inhibitor Mk2206 did not block hypercapnia-induced activation of the mTORC1-target S6K1 in IAV-infected cells. This suggests that mTORC1 is activated independently of Akt in our system, possibly by the viral M2 protein, which was augmented by hypercapnia, and is known to promote mTORC1 activity [59]. The mTORC1/mTORC2 inhibitor pp242 also blocked Akt activation in IAV-infected cells, suggesting that hypercapnia-induced Akt activation is mediated by mTORC2, which is known to phosphorylate Akt [60]. Further, Mk2206 blocked the hypercapnia-induced increases in expression of SREBP2, HMGCS1, and viral NP, and IAV replication as well, indicating that effects of elevated CO_2_ on cholesterol accumulation and viral proliferation in bronchial epithelial cells are linked to Akt. It is also notable that statins can inhibit the Akt/mTOR pathway [61], so in addition to blocking hypercapnia-induced IAV replication by decreasing cholesterol synthesis, statins may also inhibit CO_2_ effects by interfering with Akt and mTOR signaling.

In conclusion, our findings suggest that pharmacologic inhibition of SREBP2, enzymes required for cholesterol biosynthesis, or the mTOR/Akt signaling pathway, may hold promise for reducing susceptibility to and/or improving outcomes of influenza infection in patients with advanced lung disease and hypercapnia.

## 4. Materials and Methods

### 4.1. Materials

All materials were purchased from Sigma-Aldrich, St. Louis, MO, USA; unless otherwise specified.

### 4.2. Mice

C57Bl/6 mice of 6 to 10 weeks of age and from Jackson Laboratories were used. Experiments were performed according to protocols approved by the Institutional Animal Care and Use Committee of Northwestern University and according to National Institutes of Health guidelines for the use of rodents.

#### Exposure of Mice to Hypercapnia and Influenza A Virus Infection

Mice were exposed to normoxic hypercapnia (10% CO_2_/21% O_2_/69% N_2_) in a BioSpherix A environmental chamber (BioSpherix, Lacona, NY, USA). O_2_ and CO_2_ concentrations in the chamber were maintained at the indicated levels using ProOx C21 O_2_ and CO_2_ controllers (BioSpherix). As controls in all experiments, age-matched mice, simultaneously maintained in air, were used. Mice pre-exposed to air or hypercapnia for 3 days were anesthetized with isoflurane and intubated with a 20-gauge Angiocath™ catheter (Becton, Dickinson and Company, Franklin Lakes, NJ, USA). Mice were inoculated intratracheally with IAV (A/WSN/33 [H1N1]), a mouse-adapted strain, kindly provided by Robert Lamb, Ph.D., Sc.D., Northwestern University, Evanston, IL, or with PBS as control, as previously described [16], and returned to their previous air or hypercapnia exposure. Mice were infected with 30 pfu/mouse in 50 μL of PBS. Animals were sacrificed at 1 and 4 dpi (Figure 1A). MK2206 (120 mg/kg body weight), or the drug vehicle captisol (MedChemExpress, Monmouth Junction, NJ USA) was administrated by oral gavage at 1 d before HC exposure and 2 days after HC exposure. Mice were sacrificed after 7 days of HC exposure (Figure 5B) or infected with IAV, as before, after 3 days of HC exposure and sacrificed at 7 dpi (Figure 5D). Mice infected with IAV were weighed daily and monitored every 8 h for development of severe distress (slowed respiration, failure to respond to cage tapping, failure of grooming, and fur ruffling). We previously showed that air-breathing mice infected with IAV at 30 pfu all survived, while those exposed to 10% CO_2_ exhibited 100% mortality by 10 dpi [16]. In addition, after IAV infection, mice lost weight at the same rate in air and 10% CO_2_, but IAV-induced inflammatory lung injury was greater in hypercapnic mice than in air-breathing mice [16].

### 4.3. Cells

Primary HBE cells (LONZA, Bend, OR, USA) were cultured on PneumaCult-Ex Plus media (StemCell, Cambridge, MA, USA). After 70–80% confluency, cells were dissociated with Animal Component-Free Cell Dissociation Kit (StemCell) and seeded on Transwells (Corning, Corning, NY, USA) coated with Collagen type IV (0.3 mg/mL, Sigma-Aldrich). When cells reached confluency, they were exposed to ALI, and the basal media was replaced with PneumaCult-ALI medium (StemCell). Experiments were performed upon full cell differentiation (∼3–4 wk on air) as assessed by visual confirmation of beating cilia and mucus [62]. BEAS-2B cells, a SV-40-transformed human bronchial epithelial cell line (ATCC CRL-9609), were cultured in DMEM: F12, 5% FBS media. Cells were incubated at 37 °C, 5% CO_2_, and all media contain penicillin (100 U/mL) and streptomycin (100 μg/mL).

#### Exposure of Cells to Normocapnia and Hypercapnia and Infection with IAV

Cells were exposed to normocapnia (5% CO_2_, PCO_2_ 36 mmHg/95% air) or normoxic hypercapnia (15% CO_2_, PCO_2_ 108 mmHg)/21% O_2_/64% N_2_, in a C-174, Biospherix environmental chamber). This chamber was contained within the same incubator where control cultures were simultaneously exposed to normocapnia. Before addition to cultures, media were pre-saturated with 5% or 15% CO_2_. Cells were pre-exposed to normocapnia or hypercapnia for 2 days, and IAV (MOI 1) was added for 1 h. Then, the cells were washed and cultured for an additional day in normocapnia or hypercapnia, respectively.

### 4.4. Immunofluorescence Microscopy in Tissue Sections and Cell Cultures

Mice were euthanized, lungs were HBSS perfused via the right ventricle, and a 20-gauge angiocath was sutured into the trachea. Lungs and heart were removed en bloc, and lungs were inflated with 0.8 mL of formalin at a pressure of 16 cm H_2_O. Tissue was embedded in paraffin, and 5-μm sections were then deparaffinized with xylene, rehydrated by using graded ethanol, and subjected to antigen retrieval using sodium citrate buffer (10 mM, pH 6.0), as before [16]. Tissues were labeled with anti-NS1 (GeneTex, Irvine, CA, USA; GTX125990), anti-M2 (Invitrogen, Irvine, CA, USA; MA1-082), anti-ABCA1 (Thermo Fisher Scientific, Waltham, MA, USA; MA5-16026), anti-SREBP2 (Novus, Centennial CO, USA; NBP1-54446SS), or anti HMGCS1 (Abcam, Waltham, MA, USA; ab87246) antibodies followed by Alexa-conjugated secondary antibodies (1 μg/mL). Cells were fixed with 4% PFA for 15 min, permeabilized with 0.1% Triton X100 for 5 min, and labeled with anti-NS1, anti NP (Abcam, ab128193), or anti-anti-p^S473^Akt (CellSignal, 4060S) antibodies followed by Alexa-conjugated secondary antibodies. Cells were co-labeled with acetylated tubulin (cilliated cell marker) using anti-acetylated-tubulin (T7451) antibody, followed by Alexa-conjugated antibody (1 μg/mL). DAPI was used to visualize nuclei in all cases, and Gel/Mount (Biomeda, San Jose, CA, USA) was used to mount the slides. Of note, mouse or rabbit IgGs were used as a nonimmune staining control that was negative for all protocols. Images were obtained using the same exposure time for all samples from a given experimental set using Axiovert 200M Fluorescence Microscope (Zeiss, Buffalo Grove, IL, USA). Exposure time was selected based on the brightest stained sample to avoid saturation and used for all other samples in the set, resulting in equal subtraction of background autofluorescence from all sets. NS1- and M2-positive cells and nuclei in the airways were assessed in at least 3 airways from 3 to 4 different lungs from mice from 2 to 3 independent experiments using a pipeline adapted from the IdentifyPrimaryObjects module of CellProfiler 4.2.6 cell image analysis software (Broad Institute Inc., Cambridge, MA, USA) [63]. Adaptive threshold strategy and Otsu thresholding method were applied. Results were expressed as percentage of NS1- or-M2 positive cells. HMGCS1 and SREBP2 fluorescence intensity was quantified using NIH ImageJ software version 1.54g from selected airways. Nuclei/field were assessed with CellProfiler. HMGCS1 and SREBP2 fluorescence intensity was expressed in arbitrary units (AUs)/cell. Cultured HBE and BEAS-2B cells labeled for NS1, acetylated tubulin, HMGCS1, pAKt, and NP protein were also imaged using IF microscopy (3 fields/condition from 4 independent experiments). pAkt fluorescence intensity was quantified using NIH ImageJ software. Nuclei/field were assessed with CellProfiler cell image analysis software. pAkt fluorescence intensity was expressed in arbitrary units (AU)/cell. NP-positive cells were assessed using CellProfiler as above.

### 4.5. Immunoblotting

The presence of indicated proteins in cell homogenates was assessed by immunoblotting using the following Abs: anti-NS1, anti-ABCA1, anti-SREBP2, anti-p^S473^Akt, anti-p ^Thr389^-p70 S6 Kinase (Cell Signal, 9234T), or anti-βactin (Abcam, ab8226). Signals were detected following incubation with IRDye (1:10,000, LI-COR, Lincoln, NE, USA) Biosciences or HRP-conjugated (1:5000) secondary Abs for 1 h at room temperature using the LI-COR Odyssey Fc Imaging System. Membranes were developed and densitometry was performed using ImageStudio™ software version 5.2 (LI-COR).

### 4.6. Viral Adhesion and Internalization

Viral adhesion and internalization were determined as before [64]. Briefly, to monitor viral adhesion, IAV (20 MOI) was bound on ice for 90 min to cells pre-exposed to normocapnia or hypercapnia. The cells were washed, fixed, and processed as described above for NP IF. To measure virus internalization, cells pre-exposed to normocapnia or hypercapnia were incubated with IAV on ice during 90 min and either fixed immediately or maintained at 37 °C for 30 min. The cells were then washed with 0.1 M glycine-0.1 M NaCl, pH 3.0, buffer for 2 min to remove non-internalized virus; fixed; and permeabilized. IAV localization was assessed using anti-nucleoprotein (NP) antibodies. Cell images (3 fields per condition from at least 4 independent experiments) were obtained using the same exposure time for all samples from a given experimental set using Axiovert 200M Fluorescence Microscope (Zeiss). Exposure time was selected based on the brightest stained sample to avoid saturation and used for all other samples in the set, resulting in equal subtraction of background autofluorescence from all sets. NP fluorescence intensity was quantified using NIH ImageJ software. Nuclei/field were assessed with CellProfiler cell image analysis software. NP fluorescence intensity was expressed in arbitrary units (AU)/cell.

### 4.7. Cell Cholesterol

Cells were lysed using RIPA buffer, and cholesterol was measured using Amplex red assay following manufacturer’s protocol. Cholesterol concentration was expressed as µg/mg protein.

### 4.8. Viral Plaque Assay

Quantification of infectious virions was performed by viral plaque assay. Briefly, cell supernatants were centrifuged (4 °C, 2000 rpm for 10 min). MDCK cells were grown in 6-well plates to 100% confluence and then incubated with serial 10-fold dilutions of lung homogenate in DMEM and 1% bovine serum albumin (BSA) for 1 h (37 °C). Supernatants were then aspirated, the cells were washed with PBS, 3 mL of replacement media [2.4% Avicel (IMCD, Harrington Park, NJ, USA), 2× DMEM, and 1.5 μg of N-acetyl trypsin] was added to each well, and the plates were incubated for 3 days. The overlay was then removed, and viral plaques were visualized using naphthalene black dye solution (0.1% naphthalene black, 6% glacial acetic acid, 1.36% anhydrous sodium acetate). Results were expressed as pfu.

### 4.9. Statistics

Statistical analyses were carried out using Prism software (GraphPad Prism 10.4.0). Data are presented as means ± SD. Differences between two groups were assessed using a Student’s *t*-test. Levene’s test was used to analyze the homogeneity of variances. Differences between multiple groups were assessed by ANOVA followed by Sidak’s multiple comparisons test.

## Data Availability

The original contributions presented in the study are included in the article/Supplementary Materials; further inquiries can be directed to the corresponding author.

## Supplementary Materials

The following supporting information can be downloaded at: https://www.mdpi.com/article/10.3390/ijms26094133/s1.
