# Supplementary material for: Hypercapnia Increases Influenza A Virus Infection of Bronchial Epithelial Cells by Augmenting Cellular Cholesterol via mTOR and Akt
Source: Int J Mol Sci. 2025 Apr 26;26(9):4133. doi: 10.3390/ijms26094133 (PMC12071803; doi:10.3390/ijms26094133)
Supplement: Supplementary file 1 [file ijms-26-04133-s001.zip › ijms-3269612-supplementary.pdf]

Supplementary Material

Hypercapnia Increases Influenza A Virus Infection of Bronchial Epithelial Cells by Augmenting Cellular Cholesterol via mTOR and Akt

Fei Chen[1], Aiko Matsuda[1], Peter H. S. Sporn[1, 2, †], S. Marina Casalino-Matsuda[1, *, †]

[1] Division of Pulmonary and Critical Care Medicine, Feinberg School of Medicine, Northwestern University, Chicago, IL, 60611, USA
[2] Research Service, Jesse Brown Veterans Affairs Medical Center, Chicago, IL, 60612, USA
* Correspondence: marinamatsuda@northwestern.edu
† These authors contributed equally to this work.

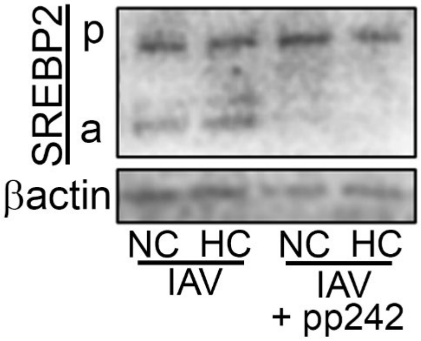

Figure S1. Inhibition of mTOR blocks hypercapnic activation of SREBP2. BEAS-2B were exposed to NC or HC for 18 h, then infected with IAV for an additional 18 h in the absence or presence of the mTOR inhibitor pp242 (1 μM). Cleavage of pre-SREBP2 (p) to its active form (a) was assessed by immunoblot. βactin was used as loading control.

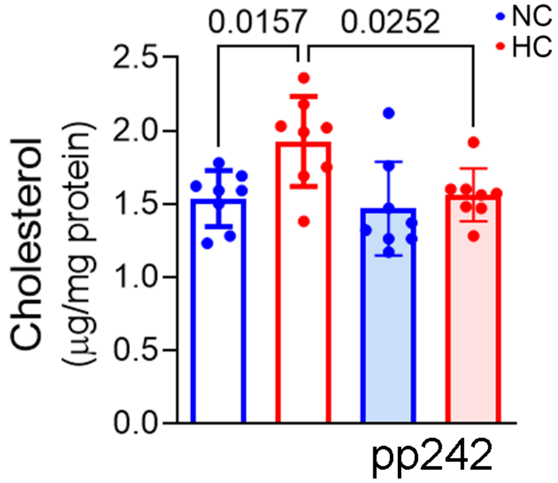

Figure S2. Inhibition of mTOR blocks increased cellular cholesterol induced by hypercapnia. BEAS-2B were exposed to NC or HC for 18 h in the absence or presence of the mTOR inhibitor pp242 (1 µM) after which cellular cholesterol was measured using Amplex Red assay. Individual data points, means ± SEM and P values are shown.

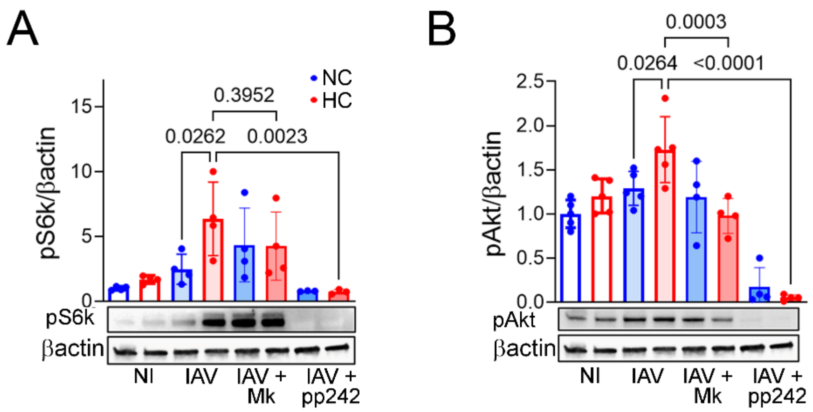

Figure S3. Inhibition of mTOR pathway blocks Akt and S6K phosphorylation induced by hypercapnia and IAV while inhibition of Akt only blocks Akt activation. BEAS-2B cells were exposed to NC or HC for 2 h, then infected with IAV for an additional 2 hours in the absence or presence of the mTOR inhibitor pp242 (1 μM) or the Akt inhibitor MK2206 (MK, 5 μM). Phosphorylation of signaling proteins pAkt (A) and pS6k (B) were assessed by immunoblot. βactin was used as loading control. Individual data points, means ± SEM and P values are shown (A-B).
